# Multimodal reasoning based on knowledge graph embedding for specific diseases

**DOI:** 10.1093/bioinformatics/btac085

**Published:** 2022-02-12

**Authors:** Chaoyu Zhu, Zhihao Yang, Xiaoqiong Xia, Nan Li, Fan Zhong, Lei Liu

**Affiliations:** Institute of Biomedical Sciences and School of Basic Medical Science, Shanghai Medical College, Fudan University, Shanghai 200032, China; College of Computer Science and Technology, Dalian University of Technology, Dalian 116024, China; Institute of Biomedical Sciences and School of Basic Medical Science, Shanghai Medical College, Fudan University, Shanghai 200032, China; College of Computer Science and Technology, Dalian University of Technology, Dalian 116024, China; Institute of Biomedical Sciences and School of Basic Medical Science, Shanghai Medical College, Fudan University, Shanghai 200032, China; Institute of Biomedical Sciences and School of Basic Medical Science, Shanghai Medical College, Fudan University, Shanghai 200032, China; Jihua Laboratory, Engineering Research Center for Intelligent Robotics, Guangzhou 510000, China

## Abstract

**Motivation:**

Knowledge Graph (KG) is becoming increasingly important in the biomedical field. Deriving new and reliable knowledge from existing knowledge by KG embedding technology is a cutting-edge method. Some add a variety of additional information to aid reasoning, namely multimodal reasoning. However, few works based on the existing biomedical KGs are focused on specific diseases.

**Results:**

This work develops a construction and multimodal reasoning process of Specific Disease Knowledge Graphs (SDKGs). We construct SDKG-11, a SDKG set including five cancers, six non-cancer diseases, a combined Cancer5 and a combined Diseases11, aiming to discover new reliable knowledge and provide universal pre-trained knowledge for that specific disease field. SDKG-11 is obtained through original triplet extraction, standard entity set construction, entity linking and relation linking. We implement multimodal reasoning by reverse-hyperplane projection for SDKGs based on structure, category and description embeddings. Multimodal reasoning improves pre-existing models on all SDKGs using entity prediction task as the evaluation protocol. We verify the model’s reliability in discovering new knowledge by manually proofreading predicted drug–gene, gene–disease and disease–drug pairs. Using embedding results as initialization parameters for the biomolecular interaction classification, we demonstrate the universality of embedding models.

**Availability and implementation:**

The constructed SDKG-11 and the implementation by TensorFlow are available from https://github.com/ZhuChaoY/SDKG-11.

**Supplementary information:**

Supplementary data are available at *Bioinformatics* online.

## 1 Introduction

Knowledge Graph (KG) is a way to store knowledge and reveal the dynamic development law of a field. KG represents facts in the real-world through a large number of triplets (head entity, relation and tail entity), denoted as (h, r, t). Large integrated KGs like Freebase (Bollacker, 2008) and DBpedia (Lehmann *et al.*, 2015) keep expanding. They have been successfully used in many applications, such as recommendation systems (Wang *et al.*, 2019a) and question answering (Huang *et al.*, 2019).

In the field of biomedicine, the application of KG is becoming increasingly popular for its specialized knowledge that only domain experts can understand well (Mohamed *et al.*, 2021). The influential roles of KG in predicting protein drug targets Mohamed *et al*. (2020) and adverse drug reactions (Zhang *et al.*, 2021) are both convincing examples. The triplets of biomedical KGs can be manually filled out by experts or automatically extracted from Electronic Medical Records (EMRs) and literature (Li *et al.*, 2020). The former is labor-intensive for large-scale KGs; While the latter is becoming more effective benefit from the rapid improvement of natural language processing.

Most existing biomedical KGs focus on particular subfields, such as DrugBank (Wishart *et al.*, 2018) for drugs and UniProt (UniProt Consortium, 2019) for proteins. However, these subfields are divided at the entity level, and few KGs focus on specific diseases. Specific Disease Knowledge Graph (SDKG) mainly focuses on the knowledge of a particular disease, which can play a more professional role in guiding the causes, treatments and prognoses of the disease. Recently, in response to the COVID-19, over three associated SDKGs had been constructed for drug repurposing (Al-Saleem *et al.*, 2021; Che *et al.*, 2021; Zhang *et al.*, 2021). A chronic obstructive pulmonary disease (COPD) SDKG was established to assist in diagnosing early curable stage COPD (Fang *et al.*, 2019). There was also a melanoma SDKG built to support precision medicine (Kang *et al.*, 2020). Considering the event that it is hard to access all the knowledge from literature (e.g. all PubMed abstracts), limiting to several diseases allows for a greater concentration of valid information. We consider 11 diseases in this work, named SDKG-11, including 5 cancers (colon cancer, gallbladder cancer, gastric cancer, liver cancer and lung cancer) and 6 non-cancer diseases (Alzheimer’s disease, COPD, coronary heart disease, diabetes, heart failure and rheumatoid arthritis). The morbidity and mortality of these diseases are significantly high, seriously threatening people’s lives (WHO, 2016). Especially, lung cancer, colon cancer, liver cancer and gastric cancer ranked the top four in the global cancer mortality rate in 2020 (Sung *et al.*, 2021).

Since new biomedical knowledge is being presented every day, almost all constructed biomedical KGs are incomplete (Nickel *et al.*, 2016). In addition to the methods mentioned above, new knowledge can also be reasoned by the existing knowledge. Knowledge Graph Embedding (KGE) has recently emerged as a paradigm for KG reasoning (Alshahrani *et al.*, 2021; Wang *et al.*, 2017). KGE maps entities and relations into a low-dimensional vector space, using simple mathematical calculations instead of explicitly defining the reasoning process, improving computational efficiency vastly. KGE model defines a scoring function f(h,r,t) to measure the probability of the existence of a triplet (Bordes *et al.*, 2013). To improve the reasoning effectiveness, some models aim to strengthen the expressive ability of the scoring function (Nguyen *et al.*, 2018; Wang *et al.*, 2014), and some multimodal models add additional information, such as categories (Xie *et al.*, 2016) and descriptions (Nie and Sun, 2019).

This study proposes a complete SDKG construction and multimodal reasoning process. Firstly, we constructed the original SDKGs from biomedical literature. Secondly, we built the standard entity set from specialized biomedical databases. Then, we refined the original SDKGs by entity linking and relation linking to obtain SDKG-11. Finally, we reasoned on the SDKGs by multimodal KGE model. To verify the reliability of inferential knowledge, we manually proofread the predicted drug–gene, gene–disease and disease–drug pairs. To demonstrate the universality of embedding results, we served them as pre-trained knowledge for biomolecular interaction classification.

## 2 Materials and methods

### 2.1 Original triplet extraction

Based on the aliases of 11 selected diseases (Supplementary Appendix S1), we extract triplets from the titles, running titles, keywords, abstracts and conclusions of PubMed indexed literature published between 1980 and 2020. We only consider journals with an impact factor no <2.0 of the year 2020.

Triplet extraction has two main steps: Named Entity Recognition (NER) Wang *et al.* (2019b) and Relation Extraction (RE) (Sangrak and Kang, 2018). NER identifies biomedical entities from literature texts, and we use Att-BiLSTM-CRF (Luo *et al.*, 2018) to accomplish that. RE extracts the relation among these entities identified by the NER, which is performed by a combination of BiLSTM (Kiperwasser and Goldberg, 2016) and ResNet (He *et al.*, 2016).

**Fig. 1.**
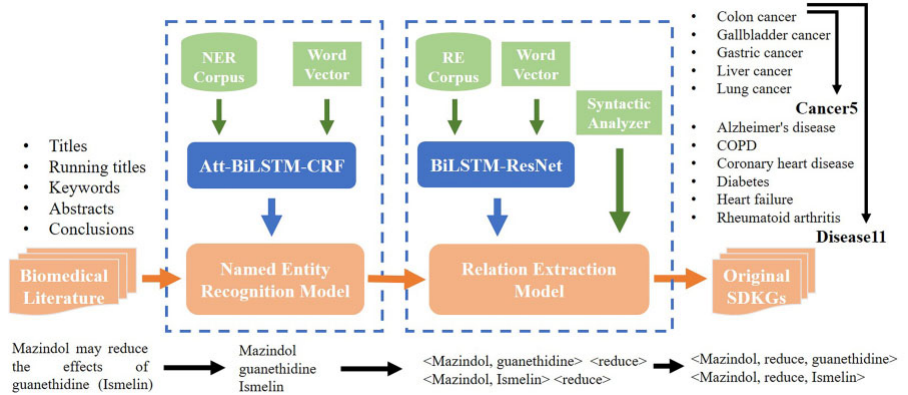
Flow chart of original triplet extraction. An example is shown in the bottom of the figure

We integrate all the extracted triplets of each specific disease as original SDKG, combine the five cancers as a Cancer5 KG, and build a Disease11 KG consisting of all the 11 diseases. In addition, we record the complete sentence of each original triplet’s provenance for subsequent processing. Figure 1 is the flow chart for this section.

### 2.2 Standard entity set

**Fig. 2.**
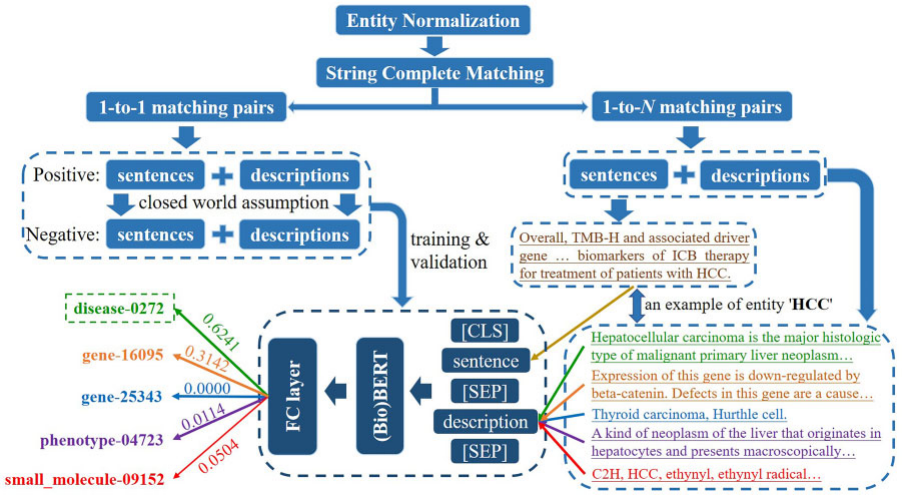
The structure and an example of entity linking. The [CLS] and [SEP] symbols are the start and delimiter tokens in the (Bio)BERT. The original entity ‘HCC’ matches to five standard entities (‘disease-0272’, ‘gene-16095’, ‘gene-25343’, ‘phenotype-04723’ and ‘small_molecule-09152’) by entity normalization and string complete matching. The brown sentence is the text that extracted the ‘HCC’ entity, while the green, orange, blue, purple and red sentences are the description annotations of the five standard entities. They are input into the trained model, and the result shows that this ‘HCC’ entity corresponds to ‘disease-0272’ with the highest score 0.6241

Biomedical entity names are easily plagued by synonymy and polysemy phenomena, which will increase the unreliability and redundancy of KG. For the former, ‘HCC’ and ‘liver cancer’ may both mean the entity ‘hepatocellular carcinoma’. This step builds a standard entity set containing all the synonymies so that the same entities can point to a unique node. For the latter, besides denoting a disease, ‘HCC’ also corresponds to two genes, a phenotype, and a small molecule (Fig. 2). It will be addressed by entity disambiguation in Section 2.3.1.

There are 165 062 biomedical entities in the standard entity set (Table 1), all of which are extracted from their specialized databases. Among them, genes are extracted from NCBI-Gene (Brown *et al.*, 2015), miRNAs from MirBase (Kozomara *et al.*, 2019), proteins from UniProt (UniProt Consortium, 2019), small molecules from ChEBI (Janna *et al.*, 2016), drugs from DrugBank (Wishart *et al.*, 2018), phenotypes from HPO (Köhler *et al.*, 2019) and diseases from OMIM (Amberger *et al.*, 2015). We encode all entities with type-serial numbers like ‘gene-00001’. We also extract their functional annotations (category and description) to support subsequent multimodal reasoning.

**Table 1.** Statistic of standard entity set

Entity type	Number	Number of category	Description coverage (%)
Gene	44 570	5	35.77
miRNA	4650	2	00.00
Protein	21 722	4	91.31
Small molecule	57 130	0	83.11
Drug	13 790	3	60.02
Phenotype	13 692	0	78.02
Disease	9508	1	85.01

*Note*: The last two columns indicate the number of category annotations and non-empty character percentage of description annotations for each entity type.

Category annotations are particular for each entity type. For example, the category annotation of a protein includes its status (‘Experimental evidence at protein level’, etc.) and its Gene Ontology (The Gene Ontology Consortium, 2019) annotations. Besides, entity type (gene, disease, etc.) is treated as a particular category. The detailed category annotations are provided in Supplementary Appendix S2.

Description annotations consist of multiple text contents concatenated in order of importance. For example, the description annotation of a disease is attached by summary, clinical features, molecular genetics, mapping and inheritance texts. As some descriptions are empty characters, we use the splice of all synonymies instead. The detailed description annotations are provided in Supplementary Appendix S3.

### 2.3 Specific disease KG construction

#### 2.3.1 Entity linking

Original triplets extracted from literature should be linked to the standard entity set. Firstly, we perform entity normalization for original entities and synonymies in the standard entity set, including outlier screening, token stem processing and token reorder. Then, we link original entities to the standard entity set with the principle of string complete matching. Since a synonymy may appear in multiple standard entities, we build an end-to-end entity disambiguation model to select the most suited standard entity for 1-to-*N* mapping pairs.

Using the contexts of original entities (i.e. complete sentences) and the contexts of standard entities (i.e. description annotations) as inputs, the disambiguation model outputs the matching score through an encoder and a Fully Connected layer. We serve all 1-to-1 mapping pairs as the positive set and generate a negative set of equal numbers for them. The combination of these two sets is then randomly divided into a training set (90%) and a validation set (10%).

We apply a pre-training language model BERT (Devlin *et al.*, 2019), and its biomedical version BioBERT (Lee *et al.*, 2019), as encoders, respectively. They both perform fine-tuning by 10 epochs, and their accuracies are checked on the validation set. Figure 2 is the structure and an example of this step. More information about the pre-training language models and the complete training details are provided in Supplementary Appendices S4 and S5.

#### 2.3.2 Relation linking

The main problems of biomedical relations are noises and synonymy phenomena, so we perform relation normalization for all original relations. Besides outlier screening and token stem processing, we also do part-of-speech tagging and keep only nouns, verbs and adverbs. Unlike entities, relations are not numerous, and lack a standard set to match. Therefore, we build a mapping dictionary manually for frequently occurring relations with reference to a built relation hierarchy structure (Zhao *et al.*, 2019). For instance, the relations ‘related’ and ‘correlated’ are both represented by the relation ‘associate’.

### 2.4 Knowledge graph embedding

We build the multimodal reasoning model by three parts: structure embedding (S), category embedding (C) and description embedding (D). For each SDKG, we randomly divide it into a training set (80%), a validation set (10%) and a test set (10%), ensuring that all entities and relations have appeared in the training set.

#### 2.4.1 Structure embedding

We apply three representative models, TransE (Bordes *et al.*, 2013), TransH (Wang *et al.*, 2014) and ConvKB (Nguyen *et al.*, 2018) as structure embedding parts, respectively.

TransE believes that if a triplet exists, its vector representations should conform to: h+r≈t. Its structure embedding is defined as:

$$S_{\mathrm{TransE}}\left(h,r,t\right)=h+r-t.$$

Despite its simplicity and efficiency, TransE cannot handle non-1-to-1 relations. TransH introduces a hyperplane normal vector $w_{r}$ for each relation r so that each entity has a different vector representation facing foreign relations. While ConvKB incorporates the principle of TransE: h+r-t, and the convolution operation makes the model more parameter-efficient. Their structure embeddings are defined as:

$$\begin{array}{c} S_{\mathrm{TransH}}(h,r,t)=(h-w_{r}^{T}hw_{r})+r-(t-w_{r}^{T}tw_{r}),\\ S_{\mathrm{ConvKB}}(h,r,t)=\mathrm{ReLU}\left(\left[h;r;t\right]\times \Omega \right), \end{array}$$

where matrix [h;r;t] is the concatenation of a triplet, * is the convolution operator, Ω is the concatenate of filters (initialized as a 1×3 vector [0.1, 0.1, -0.1]) and $\mathrm{ReLU}\left(x\right)=\max(x,0)$.

#### 2.4.2 Category embedding

We first randomly initialize a category embedding matrix with the same dimension as structure embedding, which will be learned jointly with structure embeddings. Then we take the mean value of embedding vectors of entity *e*’s all categories as its category embedding:

$$e_{c}=\frac{1}{\left|e^{c}\right|}\sum_{c\in e^{c}}c,$$

where $e^{c}$ is the category set of *e*. The category embedding of a triplet is defined as:

$$C\left(h,r,t\right)=h_{c}-t_{c}.$$

#### 2.4.3 Description embedding

We use BioBERT to convert description annotations into computable vectors. The description embedding of entity e is defined as:

$$e_{d}={W_{D}}^{T}\mathrm{BioBERT}\left(e^{d}\right),$$

where $e^{d}$ is the description annotation of e, and $W_{D}$ is a weight matrix. We train the description embedding of all entities in advance by 10 fine-tuning epochs and fixed them as characteristic inputs. The description embedding of a triplet is defined as:

$$D\left(h,r,t\right)=h_{d}-t_{d}.$$

#### 2.4.4 Multimodal learning

**Fig. 3.**
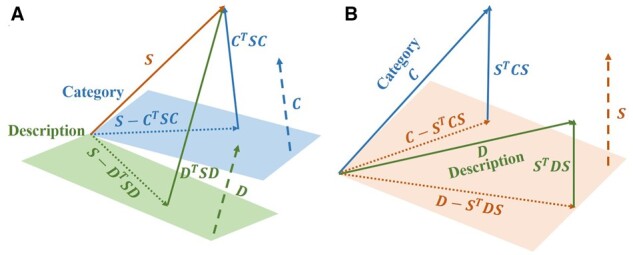
The schematic of (**A**) hyperplane and (**B**) reverse-HP. Orange for structure, blue for category and green for description one. The dashed lines represent normal vectors, which should be normalized to unit normal vectors

**Fig. 4.**
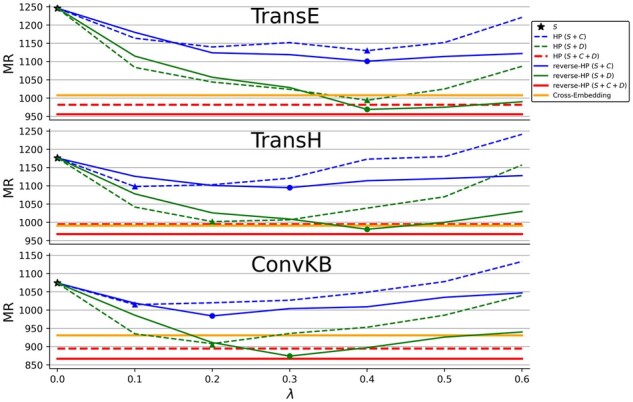
Comparison of multimodal learning methods. Blue, green and red lines represent *S + C*, *S + D* and *S + C+D* configurations. HP and reverse-HP are denoted by dotted and solid lines, respectively. The yellow line represents cross-embedding. Triangles and dots represent the optimal $\lambda^{*}$ for that annotation.

Cross-embedding (Tang *et al.*, 2019; Xie *et al.*, 2016) and hyperplane projection (HP) (Guan *et al.*, 2019; Xiao *et al.*, 2017) are two traditional multimodal learning methods. The cross-embedding scoring function for TransE is defined as:

$$f\left(h,r,t\right)=-{\left|\left|h-r+t\right|\right|}_{2}^{2}-\sum_{M\in \left\{C,D\right\}}\left({\left|\left|h_{M}-r+t\right|\right|}_{2}^{2}+{\left|\left|h-r+t_{M}\right|\right|}_{2}^{2}\right),$$

and similar formulas for TransH and ConvKB. In the HP method, category and description embeddings are regarded as two normal vectors (Fig. 3A). However, we believe that structure embedding should be the core part of multimodal learning, since it contains essential knowledge from literature. Hence, we propose reverse-hyperplane projection (reverse-HP), which regards the structure embedding as the hyperplane (Fig. 3B). On the one hand, we want to minimize the module of structure embedding vector, which is consistent with the original intention of structure embedding; on the other hand, we wish to maximize the projections of category and description embeddings on structure hyperplane, that fully extract the meanings of annotations. The results of this work are based on reverse-HP after comparison (Fig. 4).

For TransE and TransH, the final scoring functions of reverse-HP are defined as:

$$f\left(h,r,t\right)=-{\left|\left|S\right|\right|}_{2}^{2}+\sum_{M\in \left\{C,\;D\right\}}{\lambda_{M}\left|\left|M-{S_{*}}^{T}MS_{*}\right|\right|}_{2}^{2},$$

where $S_{*}=S/{\left||S|\right|}_{2}^{2}$, $\lambda_{C}$ and $\lambda_{D}$ are weight parameters. And the loss function is defined as:

$$L=\sum_{\left(h,r,t\right)\in G^{+},(h^{'},r^{'},t^{'})\in G^{-}}\mathrm{ReLU}(\gamma -f\left(h,r,t\right)+f\left(h^{'},r^{'},t^{'}\right)),$$

where γ is a margin hyper-parameter, $G^{+}$ represents the positive triplet set, and $G^{-}$ represents the artificially generated negative triplet set by Bernoulli trick (Wang *et al.*, 2014).

For ConvKB, the final scoring function is defined as:

$$f\left(h,r,t\right)=-{W_{S}}^{T}v\mathrm{ec}\left(S\right)+\sum_{M\in \left\{C,\;D\right\}}\lambda_{M}{W_{S}}^{T}vec\left(M-{S_{*}}^{T}MS_{*}\right),$$

where $W_{S}$ is a weight matrix, vec(·) transforms a matrix into a vector of equal elements. And the loss function is defined as:

$$L=\sum_{\left(h,r,t\right)\in G^{+}{\cup G}^{-}}\log(1+\exp\left(-y_{\mathrm{hrt}}\cdot f\left(h,r,t\right)\right)),$$

where $y_{\mathrm{hrt}}=1$ if $\left(h,r,t\right)\in G^{+}$, else $y_{\mathrm{hrt}}=-1$.

#### 2.4.5 Experimental setup

We use the Adam optimizer (Kingma and Ba, 2014) to minimize the loss function on the training set, finding the optimal hyper-parameters by grid search strategy on the validation set, evaluating the model on the test set. The search scopes of hyper-parameters are: embedding size k∈{100, 200}, margin γ∈{0.2, 0.6, 1.0}, number of filters n¯f∈{10, 20, 30}, learning rate $l¯r\in \{{5\times 10}^{-4},\;10^{-3},\;{5\times 10}^{-3}\}$, λ∈{0.0, 0.1, 0.2, 0.3, 0.4, 0.5}. In addition, we fix the batch size to be 1/40 of the training set size, and the max training epochs is 1000. All the embeddings are initialized by Glorot initialization (Glorot and Bengio, 2010) with the boundary of $\left(-\sqrt{6/k},\;\sqrt{6/k}\right)$.

The evaluation protocol of KGE models is the entity prediction task, namely, given an entity and a relation, predict another entity. Our evaluation metric is the mean rank (MR) of correct answers. Note that lower MR means better performance, and we only consider the ‘filter’ setting (Wang *et al.*, 2014). We also evaluate on PharmKG (Zheng *et al.*, 2021), a dedicated KG benchmark for biomedical data mining. Since there are no entity category and description annotations for PharmKG, we annotate the overlap part with the standard entity set and override the rest with null values.

In experiments, we consider the following four configurations:

1. *S* stands for using structure embedding only ($\lambda_{C}=0,\;\lambda_{D}=0$).
2. *S* *+* *C* stands for using structure and category embeddings ($\lambda_{C}\neq 0,\;\lambda_{D}=0$).
3. *S* *+* *D* stands for using structure and description embeddings ($\lambda_{C}=0,\;\lambda_{D}\neq 0$).
4. *S* *+* *C* *+* *D* stands for using all the three embeddings simultaneously ($\lambda_{C}\neq 0,\;\lambda_{D}\neq 0$).

### 2.5 Verification

#### 2.5.1 Statistical superiority test

To test the superiority of the selected model and configuration, we perform a multivariate Analysis of Variance (ANOVA) on the score rank. Due to the non-normal rank distribution, we employ the robust ANOVA based on median and median-of-means estimations using R package WSR2 (Mair and Wilcox, 2020). Further, one-tailed Wilcoxon tests are used as the *post hoc* tests from another perspective.

#### 2.5.2 Reliability of inferred knowledge

We perform the KG completion task on drug–gene, gene–disease and disease–drug pairs. For all the possible pairs, we calculate their scores (all relations are substituted into and retain the highest score) by the trained scoring function on each SDKG. We assume the top-scored inferred items (not in the training set) with a scale of 10% of the training set size as reliable new inferred knowledge. Then, they are combined with existent knowledge to construct comprehensive networks, which are illustrated by Cytoscape (Su *et al.*, 2014).

We further compare the disease–gene prediction result with advanced network-based methods, LINE (Tang *et al.*, 2015), Node2vec (Grover and Leskovec, 2016) and HerGePred (Yang *et al.*, 2019). Association Precision (AP) is served as an evaluation metric:

$$\mathrm{AP}=\sum_{d\in D}\left|T\left(d\right)\cap P\left(d\right)\right|/\sum_{d\in D}\left|T\left(d\right)\right|,$$

where D is the test disease set, $T\left(d\right)$ represents the test gene set of disease d and $P\left(d\right)$ is the top $\left|T\left(d\right)\right|$ predicted gene set.

In terms of clinical significance, we are especially interested in new inferred disease–drug pairs with potential clinical applications. We perform co-clustering by CoClust (Role *et al.*, 2019) for comprehensive disease–drug pairs, which is a Python package based on *K*-means clustering for one-zero variables. We set the number of clusters of each bilateral clustering as two because diseases can be divided into cancerous and non-cancerous, and the same for drugs.

#### 2.5.3 Universality of embedding models

We use embedding results as initialization parameters for the biomolecular interaction classification task, whose dataset is extracted from Pathway Commons v12 (Rodchenko *et al.*, 2019). We aim to predict the interaction in an entity pair by two steps: Step 1 to judge whether an entity pair interacts (manually generate an equal number of non-interacting entity pairs), then Step 2 to predict which kind of interaction between the interacting entities has. Finally, the overall prediction accuracy is calculated by:$\left[\mathrm{Acc}\left(\mathrm{Interacting}\right)\times \mathrm{Acc}\left(\mathrm{Step}\;2\right)+\;\mathrm{Acc}\left(\mathrm{Non}-\mathrm{interacting}\right)\right]/2$.

The initial embeddings of all entities will depend on the following five configurations: NONE stands for Glorot random initialization, while *P*, *P + C*, *P + D* and *P + C+D* stand for initialized by pre-trained embedding results of *S*, *S + C*, *S + D* and *S + C+D* configurations, respectively. Dataset description, model structure and training details are provided in Supplementary Appendix S6.

## 3 Results

### 3.1 Specific disease KGs

In the entity disambiguation step, BioBERT and BERT both converge within 10 fine-tuning epochs, showing the incredible power of the pre-training language model. And they achieve 91.3% and 90.6% accuracy on the validation set, respectively. Therefore, we use the results of entity disambiguation by BioBERT in the following analyses (Table 2). In the relation linking step, all the constructed SDKGs have 67 relations, mapped by relation hierarchy structure.

**Table 2.** Statistics of the specific disease KGs

Type	SDKG	Original		Constructed	
		#Triplet	#Entity	#Triplet	#Entity
Cancers	Colon cancer	360 695	95 837	53 858	8085
	Gallbladder cancer	36 865	13 286	4227	1585
	Gastric cancer	155 657	48 257	25 514	4854
	Liver cancer	515 371	142 564	76 723	10 186
	Lung cancer	383 582	117 338	59 262	8723
	Cancer5	1 387 710	301 460	197 009	15 258
Non-cancers	Alzheimer’s disease	159 459	43 288	22 929	4427
	COPD	30 154	11 235	4981	1615
	Coronary heart disease	101 801	29 582	14 780	3332
	Diabetes	408 433	86 341	71 036	7886
	Heart failure	104 212	36 203	17 430	4457
	Rheumatoid arthritis	129 710	36 985	17 679	3725
Disease11		2 305 019	438 993	332 937	19 416

### 3.2 Entity prediction

From MR comparisons (Table 3) and statistical analyses of robust ANOVA and *post hoc* Wilcoxon tests (Supplementary Table S1), we can observe that:

**Table 3.** MR comparisons on each constructed SDKG under three structure embedding algorithms and four configurations

Model	TransE				TransH				ConvKB			
SDKG\configuration	*S*	*S + C*	*S + D*	*S + C+D*	*S*	*S + C*	*S + D*	*S + C+D*	*S*	*S + C*	*S + D*	*S + C+D*
Colon cancer	749	644	608	**606**	708	640	**576**	587	653	604	**549**	550
Gallbladder cancer	238	230	192	**189**	225	219	197	**185**	203	174	157	**154**
Gastric cancer	474	412	377	**376**	470	417	**378**	390	390	363	338	**333**
Liver cancer	912	788	745	**735**	869	769	711	**704**	802	720	629	**627**
Lung cancer	830	702	**669**	675	805	710	663	**645**	709	646	**564**	566
Cancer5	1157	1015	900	**892**	1105	1043	926	**914**	1045	957	820	**804**
Alzheimer’s disease	433	382	354	**341**	388	360	321	**309**	352	318	293	**281**
COPD	222	194	185	**182**	210	181	**174**	**174**	175	155	145	**144**
Coronary heart disease	341	298	277	**267**	336	302	283	**270**	294	260	226	**217**
Diabetes	594	527	463	**462**	544	503	**460**	461	501	455	401	**399**
Heart failure	527	474	421	**419**	483	445	412	**411**	441	409	**348**	353
Rheumatoid arthritis	438	391	337	**336**	389	360	319	**318**	334	314	292	**291**
Disease11	1246	1101	969	**956**	1176	1095	981	**968**	1074	984	874	**867**
PharmKG	290	277	263	**255**	274	265	257	**254**	254	244	239	**236**

*Note*: The best configuration under each structure embedding algorithm is noted in bold. The best configuration for each SDKG is noted in underlining.

1. ConvKB achieves the best performance in most conditions with statistical significance.
2. Among the 14 KGs and 3 structure embedding algorithms, 35 *S* *+* *C* *+* *D* configurations achieve the best performance (0 for *S* and *S* *+* *C*, 8 for *S* *+* *D*). However, the superiority of *S* *+* *C* *+D* over *S* *+* *D* lacks statistical significance.
3. PharmKG has a comparably slight lift, since it is only partially annotated.

### 3.3 Comparison of multimodal learning

Figure 4 shows how performance (MR of the Disease11) changes by varying $\lambda_{C}$ and $\lambda_{D}$ for HP and reverse-HP, respectively, one fix at 0 when another change from 0 to 0.6 (we assume that $\lambda_{C}$ and $\lambda_{D}$ are independent, because they are the weights of two parts). We can see that HP outperforms reverse-HP initially, but reverse-HP is consistently better as λ increases. In comparing with cross-embedding, *S + C* *+* *D* configuration of both HP and reverse-HP perform better with the appropriate ${\lambda_{C}}^{*}$ and ${\lambda_{D}}^{*}$.

### 3.4 New knowledge inference

According to the criteria that 10% scale of the corresponding training set size, we finally obtain reliable new inferred knowledge of drug–gene, gene–disease and disease–drug pairs by ConvKB (*S* *+* *C* *+* *D*) (Supplementary Tables S2–S4). These results may be new discoveries that are the potential research directions. Table 4 lists the top 10 new inferred pairs of the Disease11. Respectively, 8, 9 and 9 evidences from literature can be found for 10 pairs of these three types, which fully demonstrate the reliability of our model in discovering new knowledge. And the pairs for which no evidence has been found may be some findings that beyond the scope of current knowledge.

**Table 4.** Validation of the top 10 drug–gene, gene–disease and disease–drug pairs

Pair type	Rank	Score	Head entity	Tail entity	PubMed evidence
Drug–gene	39	18.522	Interferon gamma	*IFNB1*	29 313 175
	51	17.990	Nerve growth factor	*NGF*	*Equivalent*
	54	17.874	Interferon alfa	*IL22*	30 976 912
	58	17.817	Interferon gamma	*CASP3*	22 785 177
	121	16.370	Insulin beef	*FGF21*	29 987 000
	122	16.086	Interferon kappa	*IFNG*	\
	140	16.086	Thrombin	*CD177*	\
	154	15.814	Docetaxel	*SERPINB3*	21 695 460
	159	15.534	Interferon gamma	*IFNG*	*Equivalent*
	175	15.375	Interleukin-7	*FOXP3*	32218828
Gene–disease	114	23.200	*MMP12*	Pulmonary fibrosis	33 065 600
	260	21.234	*HMGA2*	Bladder cancer	31 053 526
	452	19.706	*YAP1*	Squamous cell carcinoma	32 206 709
	565	19.159	*TIMP1*	Sarcoidosis	26 240 517
	573	19.098	*GPI*	Ovarian cancer	\
	596	19.015	*IRF8*	Autoimmune disease	30 285 234
	602	18.988	*TNFRSF25*	Lupus erythematosus	22 666 553
	645	18.789	*SATB1*	Squamous cell carcinoma	32 451 408
	654	18.755	*DLEC1*	Ovarian cancer	30 324 802
	662	18.726	*EPHA2*	Breast cancer	33 962 882
Disease–drug	37	16.939	Biliary cirrhosis	Interferon alfa	23 291 480
	128	14.023	Wilson disease	Iron	33 680 437
	151	13.373	Depressive disorder	Glutathione	26 706 022
	180	12.863	Obesity	Albumin human	22 230 555
	217	12.443	MODY	Insulin human	27 103 109
	272	11.815	MODY1	Insulin human	28 684 784
	274	11.803	Burkitt lymphoma	Fibronectin	19 625 084
	320	11.356	Pancreatic cancer	Insulin-like growth factor II	28 420 208
	356	11.026	Diabetes I	Vitamin D3	33 069 738
	383	10.744	Lamellar ichthyosis	Ethanol	\

*Note*: The Rank column also ranks the pairs in the training set. *Equivalent* means that drug and gene refer to the same biological concept, so we treat it as a correct prediction.

For the disease–gene prediction task (Table 5), all the disease–gene networks constructed by ConvKB and other three advanced network-based methods, SDKGs have relatively small AP due to domain-induced incompleteness. Nevertheless, ConvKB outperforms network-based methods, thanks to the relation embedding and multimodal annotations to compensate for network incompleteness.

**Fig. 5.**
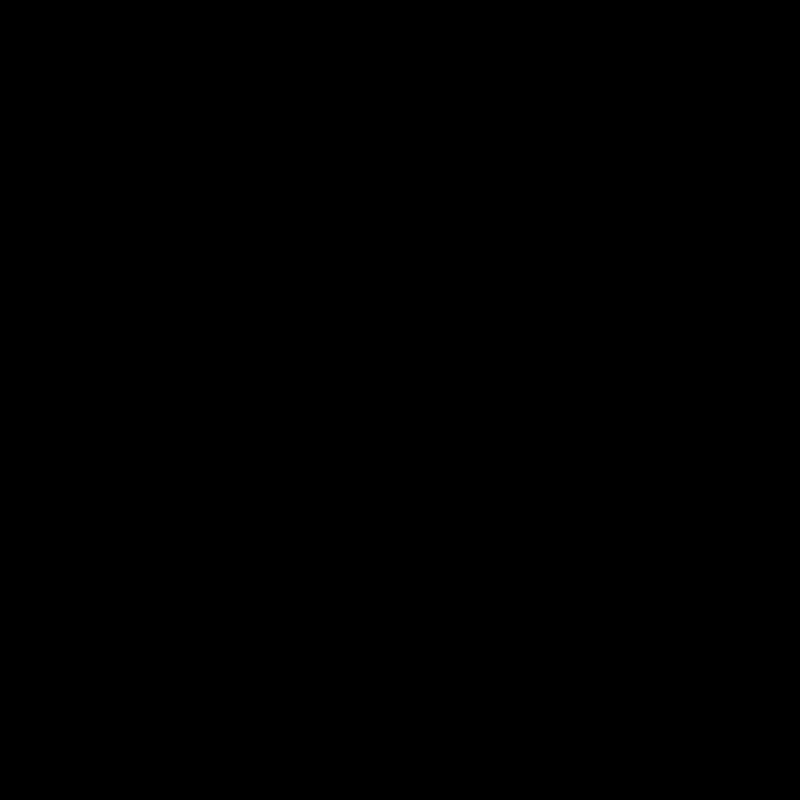
Networks contain inferred (green edges) and existent (black edges) knowledge. Yellow, red and blue nodes denote gene, disease and drug entities, respectively

Figure 5 shows the networks containing all the entities that are linked by inferred edges. The node in the center of each network is the disease itself, which is mostly connected by existent knowledge (black edges) as expected. Our model then reasons out new potential knowledge (green edges) based on this core knowledge of the specific disease field.

**Table 5.** *AP* of disease–gene prediction results based on SDKGs

SDKG\methods	LINE	Node2vec	HerGePred	ConvKB
Colon cancer	0.0492	0.0431	0.0529	**0.0683**
Gastric cancer	0.0349	0.0403	0.0430	**0.1020**
Liver cancer	0.0368	0.0314	0.0378	**0.0650**
Lung cancer	0.0132	0.0286	0.0317	**0.0782**
Alzheimer’s disease	0.0316	0.0150	0.0382	**0.1080**
COPD	0.0578	0.0751	0.0636	**0.0983**
Diabetes	0.0043	0.0281	**0.0583**	0.0540
Rheumatoid arthritis	0.0573	0.0466	0.0717	**0.1111**

*Note*: Bold number stands for the best method on that SDKG. We only list the SDKGs with $\sum_{d\in D}\left|T\left(d\right)\right|\geq 100$. ConvKB on *S + C+D* configuration.

### 3.5 Application of the inferred knowledge

**Fig. 6.**
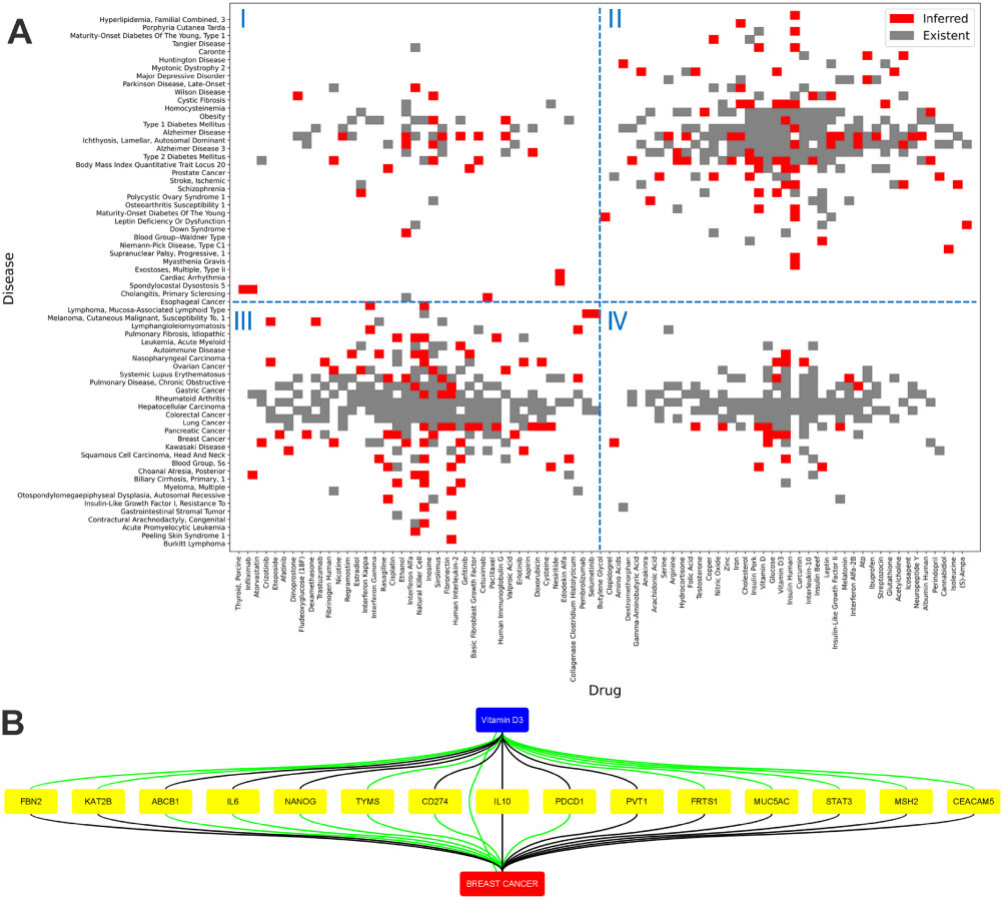
(**A**) Co-clustering result for comprehensive disease–drug pairs on the KG combining all the 11 diseases. These pairs are automatically grouped into four distinct clusters (I: anticancer drugs ∼ non-cancer diseases; II: non-anticancer drugs ∼ non-cancer diseases; III: anticancer drugs ∼ cancers; and IV: non-anticancer drugs ∼ cancers). Red and gray nodes denote inferred and existent knowledge, respectively. (**B**) A cluster of 15 {drug, gene, disease} closed-triplets with vitamin D3 and breast cancer nodes have emerged in the Cancer5 after reasoning. The inferred and existent edges are in green and black, respectively

From the drug–disease part in the Disease11 (Fig. 6A), we can observe that: in most cases, anticancer drugs are used to treat cancer and vice versa. Although non-anticancer drugs are also used extensively in cancer treatment, the reverse is not true. Most of the new inferred disease–drug pairs are in the field of anticancer drugs to treat cancer. This means expanding some anticancer drugs to more types of cancer will be the mainstream direction of drug repurposing. There are plenty of potential clinical applications of repurposing non-anticancer drugs in their original field to treat non-cancerous diseases. However, repurposing of non-anticancer drugs in carcinoma can be rarely inferred from existing knowledge. They should be mainly dependent on some subversive discoveries beyond current knowledge.

Further, we focus on {drug, gene, disease} closed-triplets for the mutual corroboration of both reliability and systematicness. The desired closed-triplet consists of each node type and contains at least one new inferred edge (Supplementary Table S5). On the one hand, we can get more evidence support from the other two edges of the triplet; on the other hand, the triplet itself is a logically closed loop that can naturally form a proposition that ‘*Gene* associates *Disease*, and *Drug* effects on *Disease* by influencing *Gene* (products)’. Take an example from the Cancer5, we discover a cluster of vitamin D3 centric closed-triplets (Fig. 6B). Vitamin D3 has been predicted to prevent or alleviate breast cancer, and the effects may work through genes, such as *IL10*. The effects of vitamin D3 on gene *IL10* (Boontanrart *et al.*, 2016) and *IL10* plays an essential role in breast cancer (Moghimi *et al.*, 2018) have both been studied. While some studies partly supported our predictions that vitamin D3 may prevent or alleviate breast cancer (Wu *et al.*, 2017) but without direct evidence.

### 3.6 Pre-trained biomolecular interaction classification

Table 6 shows the biomolecular interaction prediction result by ConvKB of the Disease11, from which we can observe that:

**Table 6.** Accuracy of pre-trained biomolecular interaction classification

Step	Relation		Configuration				
	Name	Number	NONE	*P*	*P + C*	*P + D*	*P + C+D*
**1**	Interacting	727 318	0.920	0.903	0.921	**0.922**	**0.922**
	Non-interacting	727 318	0.819	0.865	0.889	0.890	**0.891**
	Binary categories	1 454 636	0.869	0.884	0.905	0.906	**0.907**
**2**	In-complex-with	51 173	0.656	0.649	0.677	0.685	**0.697**
	Catalysis-precedes	40 343	0.919	0.886	0.916	0.919	**0.934**
	Controls-expression-of	49 707	0.855	0.861	0.908	**0.920**	0.916
	Interacts-with	148,989	0.854	0.855	0.874	0.874	**0.878**
	Controls-state-change-of	55 257	0.758	0.771	0.846	0.865	**0.870**
	Controls-phosphorylation-of	7679	0.397	0.343	0.518	**0.600**	**0.600**
	Controls-production-of	11 187	0.991	0.991	0.991	**0.995**	0.989
	Controls-transport-of-chemical	2088	0.883	0.790	0.896	0.918	**0.959**
	Chemical-affects	333 375	0.997	**0.998**	**0.998**	**0.998**	**0.998**
	Consumption-controlled-by	10 929	0.783	0.706	0.766	0.767	**0.793**
	Used-to-produce	13 118	0.803	**0.878**	0.877	0.826	0.830
	Reacts-with	3473	0.406	0.266	0.290	0.449	**0.459**
	All the 12 interactions	727 318	0.894	0.895	0.916	0.920	**0.923**
Overall			0.821	0.837	0.866	0.869	**0.871**

*Note*: Bold number stands for the best configuration on prediction.

1. Although NONE and *P* configurations have the same structure, initialized with structure embedding achieves better prediction accuracy than random initialization. It strongly supports the universality of pre-trained embedding.
2. *P* *+* *C* *+* *D* configuration has the highest prediction accuracy in all steps. It indicates that multimodal learning can further improve the performance of biomolecular interaction classification.
3. In Step 2, some interactions (controls-phosphorylation-of and reacts-with) have a rather low prediction accuracy, mainly due to their rather small sample sizes.

## 4 Discussion

The SDKG-11 is constructed based on biomedical literature, and the build process produces large-scale original triplets almost without human intervention. As a highly condensed knowledge carrier, biomedical literature contains virtually all the knowledge that has been discovered and is being studied. Thus, it should be an ideal source of triplet extraction. As for another primary source of biomedical knowledge, EMR has low overall data quality, with a lot of unforeseen noise, and varies widely across regions. However, one of the most significant advantages of EMR-based triplet extraction is that it is more clinical and real-world (Li *et al.*, 2020). Therefore, we would like to combine literature with EMR to build comprehensive SDKGs containing real-world data next.

At the model level, we evaluate TransE, TransH and ConvKB as structure embedding parts, and the experimental result shows that ConvKB is the most efficient one. The main reason is that ConvKB not only considers the transitional characteristics of TransE, but also takes advantage of the effectiveness of the convolutional neural network. If we intend to consider more advanced structure embeddings, graph neural network-based KGE model would be a solid choice due to its natural fit structure with the KG. The combination of KG and graph convolutional networks (Schlichtkrull *et al.*, 2018), as well as KG and graph attention networks (Che *et al.*, 2021; Nathani *et al.*, 2019), both have been studied. We consider that serving multimodal embedding as multiple features of the graph’s nodes would be a promising attempt.

As for multimodal learning, we apply reverse-HP rather than cross-embedding nor HP. Cross-embedding combines structure embedding with other modal embeddings, but cannot adjust the weight of each modal embedding. Previous HP models only considered description hyperplane, and their description embeddings were generated by topic model (Xiao *et al.*, 2017) and skip-gram model (Guan *et al.*, 2019). In this work, the weight parameters represent the contribution degree of each annotation with certain interpretability. Both category and description annotations can promote the inference effect (Fig. 4), because they make up the incompleteness of SDKGs to some extent, and provide new knowledge sources for structure embedding. We also observe that description annotation is better than category annotation, since the former has much more information. Moreover, when there is already description annotation (*S + D*), adding category annotation (*S + C+D*) will not improve much. Suppose, we intend to consider more modal annotations, image annotation (e.g. structure schematics of proteins and drugs) is the most likely annotation to be added (Xie *et al.*, 2017). In addition, the annotation of relation (Tang *et al.*, 2019) and multi-omics (Zheng *et al.*, 2021) are also potentially promising directions.

From the perspective of knowledge relevance, triplets from the literature of each specific disease are more focused. However, considering the completeness of information from the perspective of ‘big data’, we highly recommend combining all available information into one corpus, since some inferred knowledge from one SDKG may already exist in another SDKG. Next, we will try to extract knowledge from more general themes, such as ‘cancer’ or ‘disease’ to construct the initial KG.

## 5 Conclusion

In this work, we have proposed a complete specific disease KG construction and multimodal reasoning process. We have constructed SDKG-11, a SDKG set including five cancers, six non-cancer diseases, a combined Cancer5 and a combined Diseases11. We have evaluated our multimodal KGE model by entity prediction task and verified in some instances. We have then demonstrated the influential role of the learned embedding in the downstream biomedical task. All of the above suggests that the new knowledge, we reasoned is reliable, and the embeddings, we learned are universal. They can be helpful for research and clinical staffs in the field of some specific diseases.

## Supplementary Material

btac085_supplementary_data

## References

[btac085-B1] Al-Saleem J. et al (2021) Knowledge graph-based approaches to drug repurposing for COVID-19. J. Chem. Inf. Model., 61, 4058–4067. 34297570 10.1021/acs.jcim.1c00642

[btac085-B2] Alshahrani M. et al (2021) Application and evaluation of knowledge graph embeddings in biomedical data. PeerJ Comput. Sci., 7, e341. 10.7717/peerj-cs.341 PMC7959619 33816992

[btac085-B3] Amberger J.S. et al (2015) OMIM.org: online Mendelian Inheritance in Man (OMIM^®^), an online catalog of human genes and genetic disorders. Nucleic Acids Res., 43, D789–D798. 25428349 10.1093/nar/gku1205 PMC4383985

[btac085-B4] Bollacker K. (2008) Freebase: a collaboratively created graph database for structuring human knowledge. In: *Proceedings of the 2008 ACM SIGMOD International Conference on Management of Data, Vancouver, BC, Canada*. pp. 1247–1250.

[btac085-B5] Boontanrart M. et al (2016) Vitamin D3 alters microglia immune activation by an IL-10 dependent SOCS3 mechanism. J. Neuroimmunol., 292, 126–136. 26943970 10.1016/j.jneuroim.2016.01.015

[btac085-B6] Bordes A. et al (2013) Translating embeddings for modeling multi-relational data. In: *Proceedings of the 26th International Conference on Neural Information Processing Systems, Stateline, NV, USA*. pp. 2787–2795.

[btac085-B7] Brown G.R. et al (2015) Gene: a gene-centered information resource at NCBI. Nucleic Acids Res., 43, D36–D42. 25355515 10.1093/nar/gku1055 PMC4383897

[btac085-B8] Che M. et al (2021) Knowledge-graph-based drug repositioning against COVID-19 by graph convolutional network with attention mechanism. Future Internet, 13, 13.

[btac085-B9] Devlin J. et al (2019) BERT: pre-training of deep bidirectional transformers for language understanding. In: *Proceedings of the 57th Annual Meeting of the Association for Computational Linguistics, Florence, Italy*. pp. 4171–4186.

[btac085-B10] Fang Y. et al (2019) Diagnosis of COPD based on a knowledge graph and integrated model. IEEE Access, 7, 46004–46013.

[btac085-B11] Glorot X. , Bengio Y. (2010) Understanding the difficulty of training deep feedforward neural networks. In: *Proceedings of the Thirteenth International Conference on Artificial Intelligence and Statistics, Chia Laguna Resort, Sardinia, Italy*. pp. 249–256.

[btac085-B12] Grover A. , Leskovec J. (2016) node2vec: scalable feature learning for networks. In: *Proceedings of the 22nd ACM SIGKDD International Conference on Knowledge Discovery and Data Mining, San Francisco, USA*. pp. 855–864. 10.1145/2939672.2939754 PMC5108654 27853626

[btac085-B13] Guan N. et al (2019) Knowledge graph embedding with concepts. Knowl. Based Syst., 164, 38–44.

[btac085-B14] He K. et al (2016) Deep residual learning for image recognition. In: *2016 IEEE Conference on Computer Vision and Pattern Recognition, Las Vegas, USA*. pp. 770–778.

[btac085-B15] Huang X. et al (2019) Knowledge graph embedding based question answering. In: *Proceedings of the Twelfth ACM International Conference on Web Search and Data Mining, Melbourne, Australia*. pp. 105–113.

[btac085-B16] Janna H. et al (2016) ChEBI in 2016: improved services and an expanding collection of metabolites. Nucleic Acids Res., 44, D1214–D1219. 26467479 10.1093/nar/gkv1031 PMC4702775

[btac085-B17] Kang H. et al (2020) Building a pharmacogenomics knowledge model toward precision medicine: case study in melanoma. JMIR Med. Inform., 8, e20291. 33084582 10.2196/20291 PMC7641779

[btac085-B18] Kingma D. , Ba J. (2014) Adam: a method for stochastic optimization. Comput. Sci. arXiv: 1412.6980.

[btac085-B19] Kiperwasser E. , Goldberg Y. (2016) Simple and accurate dependency parsing using bidirectional LSTM feature representations. *Transactions of the Association for Computational Linguistics*, 4, 313–327.

[btac085-B20] Köhler S. et al (2019) Expansion of the Human Phenotype Ontology (HPO) knowledge base and resources. Nucleic Acids Res., 47, D1018–D1027. 30476213 10.1093/nar/gky1105 PMC6324074

[btac085-B21] Kozomara A. et al (2019) miRBase: from microRNA sequences to function. Nucleic Acids Res., 47, D155–D162. 30423142 10.1093/nar/gky1141 PMC6323917

[btac085-B22] Lee J. et al (2019) BioBERT: a pre-trained biomedical language representation model for biomedical text mining. Bioinformatics, 36, 1234–1240. 10.1093/bioinformatics/btz682 PMC7703786 31501885

[btac085-B23] Lehmann J. et al (2015) DBpedia - a large scale, multilingual knowledge base extracted from Wikipedia. Semant. Web, 6, 167–195.

[btac085-B24] Li L. et al (2020) Real-world data medical knowledge graph: construction and applications. Artif. Intell. Med., 103, 101817. 32143785 10.1016/j.artmed.2020.101817

[btac085-B25] Luo L. et al (2018) An attention-based BiLSTM-CRF approach to document-level chemical named entity recognition. Bioinformatics, 34, 1381–1388. 29186323 10.1093/bioinformatics/btx761

[btac085-B26] Mair P. , Wilcox R. (2020) Robust statistical methods in R using the WRS2 package. Behav. Res. Methods, 52, 464–488. 31152384 10.3758/s13428-019-01246-w

[btac085-B27] Moghimi M. et al (2018) Association of IL-10 rs1800871 and rs1800872 polymorphisms with breast cancer risk: a systematic review and meta-analysis. Asian Pac. J. Cancer Prev., 19, 3353–3359. 30583340 10.31557/APJCP.2018.19.12.3353 PMC6428528

[btac085-B28] Mohamed S.K. et al (2020) Discovering protein drug targets using knowledge graph embeddings. Bioinformatics, 36, 603–610. 31368482 10.1093/bioinformatics/btz600

[btac085-B29] Mohamed S.K. et al (2021) Biological applications of knowledge graph embedding models. Brief. Bioinform., 22, 1679–1693. 32065227 10.1093/bib/bbaa012

[btac085-B30] Nathani D. et al (2019) Learning attention-based embeddings for relation prediction in knowledge graphs. In: *Proceedings of the 57th Annual Meeting of the Association for Computational Linguistics, Florence, Italy*. pp. 4710–4723.

[btac085-B31] Nguyen D.Q. et al (2018) A novel embedding model for knowledge base completion based on convolutional neural network. In: *Proceedings of the 56th Annual Meeting of the Association for Computational Linguistics, Melbourne, Australia*. pp. 327–333.

[btac085-B32] Nickel M. et al (2016) A review of relational machine learning for knowledge graphs. Proc. IEEE, 104, 11–33.

[btac085-B33] Nie B. , Sun S. (2019) Knowledge graph embedding via reasoning over entities, relations, and text. Future Gener. Comput. Syst., 91, 426–433.

[btac085-B34] Rodchenko V.I. et al (2019) Pathway commons 2019 update: integration, analysis and exploration of pathway data. Nucleic Acids Res., 48, D489–D497. 10.1093/nar/gkz946 PMC7145667 31647099

[btac085-B35] Role F. et al (2019) CoClust: a python package for co-clustering. J. Stat. Softw., 88, 1–29.

[btac085-B36] Sangrak L. , Kang J. (2018) Chemical–gene relation extraction using recursive neural network. Database, 2018, bay060. 29961818 10.1093/database/bay060 PMC6014134

[btac085-B37] Schlichtkrull M. et al (2018) Modeling relational data with graph convolutional networks. In: *European Semantic Web Conference, Heraklion, Crete*. pp. 593–607.

[btac085-B38] Su G. et al (2014) Biological network exploration with Cytoscape 3. Curr. Protoc. Bioinformatics, 47, 8–13. 10.1002/0471250953.bi0813s47 PMC4174321 25199793

[btac085-B39] Sung H. et al (2021) Global cancer statistics 2020: GLOBOCAN estimates of incidence and mortality worldwide for 36 cancers in 185 countries. CA Cancer J. Clin., 71, 209–249. 33538338 10.3322/caac.21660

[btac085-B40] Tang J. et al (2015) Line: large-scale information network embedding. In: *International Conference on World Wide Web, New York, USA*. pp. 1067–1077.

[btac085-B41] Tang X. et al (2019) Knowledge representation learning with entity descriptions, hierarchical types, and textual relations. Inf. Process. Manag., 56, 809–822.

[btac085-B42] The Gene Ontology Consortium. (2019) The Gene Ontology Resource: 20 years and still GOing strong. Nucleic Acids Res., 47, D330–D338. 30395331 10.1093/nar/gky1055 PMC6323945

[btac085-B43] UniProt Consortium. (2019) UniProt: a worldwide hub of protein knowledge. Nucleic Acids Res., 47, D506–D515. 30395287 10.1093/nar/gky1049 PMC6323992

[btac085-B44] Wang H. et al (2019a) Knowledge graph convolutional networks for recommender systems. In: *Proceedings of the 2019 World Wide Web Conference, San Francisco, USA*. pp. 3307–3313.

[btac085-B45] Wang Q. et al (2017) Knowledge graph embedding: a survey of approaches and applications. IEEE Trans. Knowl. Data Eng., 29, 2724–2743.

[btac085-B46] Wang X. et al (2019b) Cross-type biomedical named entity recognition with deep multi-task learning. Bioinformatics, 35, 1745–1752. 30307536 10.1093/bioinformatics/bty869

[btac085-B47] Wang Z. et al (2014) Knowledge graph embedding by translating on hyperplanes. In: *Proceedings of the 28th AAAI Conference on Artificial Intelligence, Quabec City, Quebec, Canada*. pp. 1112–1119.

[btac085-B48] ΘWHO. (2016) World Health Statistics 2016: Monitoring Health for the SDGs, Sustainable Development Goals. World Health Organization, Geneva, Switzerland.

[btac085-B49] Wishart D.S. et al (2018) DrugBank 5.0: a major update to the DrugBank database for 2018. Nucleic Acids Res., 46, D1074–D1982. 29126136 10.1093/nar/gkx1037 PMC5753335

[btac085-B50] Wu Y. et al (2017) Association of vitamin D3 level with breast cancer risk and prognosis in African-American and Hispanic women. Cancers, 9, 144. 29064397 10.3390/cancers9100144 PMC5664083

[btac085-B51] Xiao H. et al (2017) SSP: semantic space projection for knowledge graph embedding with text descriptions. In: *Proceedings of the 31th AAAI Conference on Artificial Intelligence, San Francisco, USA*. pp. 3104–3110.

[btac085-B52] Xie R. et al (2016) Representation learning of knowledge graphs with hierarchical types. In: *Proceedings of the 25th International Joint Conference on Artificial Intelligence, Melbourne, Australia*. pp. 2965–2971.

[btac085-B53] Xie R. et al (2017) Image-embodied knowledge representation learning. In: *Proceedings of the 26th International Joint Conference on Artificial Intelligence, New York, USA*. pp. 3140–3146.

[btac085-B54] Yang K. et al (2019) HerGePred: heterogeneous network embedding representation for disease gene prediction. IEEE J. Biomed. Health Inform., 23, 1805–1815. 31283472 10.1109/JBHI.2018.2870728

[btac085-B55] Zhang F. et al (2021) Prediction of adverse drug reactions based on knowledge graph embedding. BMC Med. Inform. Decis. Mak., 21, 38. 33541342 10.1186/s12911-021-01402-3 PMC7863488

[btac085-B56] Zhang R. et al (2021) Drug repurposing for COVID-19 via knowledge graph completion. J. Biomed. Inform., 115, 103696. 33571675 10.1016/j.jbi.2021.103696 PMC7869625

[btac085-B57] Zhao D. et al (2019) Construct semantic type of "Gene-mutation-disease" relation by computer-aided curation from biomedical literature. In: *12th International Joint Conference on Biomedical Engineering Systems and Technologies, Prague, Czech Republic*. pp. 123–130.

[btac085-B58] Zheng S. et al (2021) PharmKG: a dedicated knowledge graph benchmark for bomedical data mining. Brief. Bioinform., 22, bbaa344. 33341877 10.1093/bib/bbaa344

