# Supplementary material for: Multimodal reasoning based on knowledge graph embedding for specific diseases
Source: Bioinformatics. 2022 Feb 12;38(8):2235–45. doi: 10.1093/bioinformatics/btac085 (PMC9004655; doi:10.1093/bioinformatics/btac085)
Supplement: btac085_supplementary_data [file btac085_supplementary_data.zip › Supplementary Appendix.docx]

**Multimodal reasoning based on knowledge graph embedding for specific diseases**

**SUPPLEMENTARY MATERIAL**

# Disease Extraction Aliases

Table 1 shows the disease search aliases when extracting original triplets. The presence or absence of case, singular or plural, and the definite article (the) are insensitive. Word order collocations can also be swapped freely.

**Table 1.** Disease search aliases.

| **Type** | **Specific Disease** | **Aliases** |
| --- | --- | --- |
| **Cancers** | Colon cancer | Carcinoma of colon  Colon carcinoma  Colorectal carcinoma  Colorectal cancer  Malignant neoplasm of colon  Malignant tumor of colon  Malignant colonic Tumor |
|  | Gallbladder cancer | Gallbladder carcinoma  Gallbladder tumor  Gallbladder neoplasm  Cancer of the gallbladder  Carcinoma of the gallbladder  Malignant tumor of the gallbladder  Malignant neoplasm of the gallbladder |
|  | Gastric cancer | Cancer of the stomach  Carcinoma of the stomach  Gastric carcinoma  Stomach cancer |
|  | Liver cancer | Cancer of the liver  Hepatic carcinoma  Hepatocellular carcinoma  Hepatoma  Liver tumor  Hepatic tumor  Liver neoplasm  Hepatic neoplasia |
|  | Lung cancer | Carcinoma of the lungs  Lung carcinoma  Neoplasm of the lung  Tumor of the lung  Pulmonary neoplasm  Cancer du poumon |
| **Non-Cancers** | Alzheimer's disease | Alzheimer disease  Alzheimer syndrome  Alzheimer dementia  Alzheimer sclerosis |
|  | Chronic obstructive pulmonary | Chronic obstructive lung disease |
|  | Coronary heart disease | Coronary disease  Coronary artery disease |
|  | Diabetes | Diabetes mellitus  Sugar diabetes |
|  | Heart failure | Heart insufficiency  Cardiac failure  Cardiac insufficiency  Myocardial failure  Congestive heart disease |
|  | Rheumatoid arthritis | Rheumatic arthritis  Atrophic arthritis  Rheumatic gout  Rheumatoid disease |

# Category Annotation

Table 2 shows the detailed category annotation for each entity type, also lists all possible values for each category annotation except for Gene Ontology (Consortium, 2019) annotations (molecular function, biological process, and cellular component). You can check out the specific content of Gene Ontology at <http://geneontology.org/>.

**Table 2.** Detailed category annotation for each entity type.

| **Entity Type** | **Category Annotation** | **Possible Values** | |
| --- | --- | --- | --- |
|  |  | **Content** | **Count** |
| Gene | chromosome | '1' ~ '22', 'X', 'Y', 'X\|Y', 'MT' | 26 |
|  | type | 'biological-region', 'ncRNA', 'protein-coding', 'pseudo', 'rRNA', 'scRNA', 'snRNA', 'snoRNA', 'tRNA' | 9 |
|  | gene ontology | \ | \ |
|  | entity type | 'gene' | 1 |
| miRNA | chromosome | '1' ~ '22', 'X', 'Y' | 24 |
|  | status | 'NAME', 'NEW', 'SEQUENCE', 'UNCHANGED' | 4 |
|  | entity type | 'mirna' | 1 |
| Protein | status | 'Experimental evidence at protein level', 'Experimental evidence at transcript level', 'Protein inferred from homology', 'Protein predicted', 'Protein uncertain' | 5 |
|  | gene ontology | \ | \ |
|  | entity type | 'protein' | 1 |
| Small molecule | entity type | 'small molecule' | 1 |
| Drug | type | 'Biotech', 'Small Molecule' | 2 |
|  | groups | 'Approved', 'Experimental', 'Illicit', 'Investigational', 'Nutraceutical', 'Vet approved', 'Withdrawn' | 7 |
|  | categories | see <https://go.drugbank.com/categories>. | 4,122 |
|  | entity type | 'drug' | 1 |
| Phenotype | entity type | 'phenotype' | 1 |
| Disease | type | 'cancer', 'non-cancer' | 2 |
|  | entity type | 'disease' | 1 |

# Description Annotation

Table 3 shows the detailed description annotation for each entity type, also lists the percentage of non-null characters for each description annotation (in order of importance).

**Table 3.** Detailed description annotation for each entity type.

| **Entity Type** | **Description Annotation** | **Percentage** |
| --- | --- | --- |
| Gene | summary | 35.77% |
| miRNA | \ | 00.00% |
| Protein | summary | 91.31% |
| Small molecule | summary | 83.11% |
| Drug | summary | 59.99% |
|  | mechanism of action | 26.72% |
|  | pharmacodynamics | 20.93% |
| Phenotype | summary | 78.02% |
| Disease | summary | 50.30% |
|  | clinical features | 74.49% |
|  | molecular genetics | 66.88% |
|  | mapping | 32.78% |
|  | Inheritance | 35.56% |

# Pre-training Language Model

There are two major classes of pre-trained language models: feature-based and fine-tuning-based. BERT (Bidirectional Encoder Representations from Transformers) is the most classic and commonly used pre-training language model. BERT was pre-trained on general domain corpora (English Wikipedia and BooksCorpus). It initializes the model with the same pre-training vectors on various tasks, and a competitive result can be achieved with only a few fine-tuning steps. Similar pre-training language models include ELMo (Peters et al., 2018), RoBERTa (Liu et al., 2019), XLNet (Yang et al., 2019), ALBERT (Lan et al., 2019), *etc.*

BioBERT has the same structure as BERT, but it was pre-trained on biomedical domain corpora (PubMed abstracts and PubMed central full-text articles). BioBERT significantly outperforms BERT on three representative biomedical text mining tasks: biomedical named entity recognition, biomedical relation extraction, and biomedical question answering.

# Entity Disambiguation Training

In the entity disambiguation step, we served all 1-to-1 mapping entity pairs as the positive set and heuristically generated a negative set of equal number for them by closed world assumption (Nickel et al., 2015). The combination of these two sets was then randomly divided into a training set (90%) and a validation set (10%) to train our model.

Applying BERT and BioBERT as encoders, respectively, their inputs contain token embedding, segment embedding, and position embedding. All embeddings have the same dimension, and any longer sentences will be truncated. After 12 self-attention layers, the outputs of encoders are 768 dimensions. The fully connected layer outputs 2 dimensions, represent the probabilities of entity pairs matched and unmatched through the softmax function, respectively.

We use the Adam optimizer to minimize the cross-entropy loss function on the training set, finding the optimal fine-tuning epochs on the validation set, ultimately specifying the optimal matches for 1-to-*N* matching entity pairs in the test set. The applied hyper-parameters are listed: length of sentence: $len\_d=150$, learning rate $l\_r=2\times{10}^{-5}$, and batch size $b\_s=15$. In addition, the max fine-tuning epochs is 10.

# Pre-trained Biomolecular Interaction Classification

There are 727,318 interactions in our biomolecular interaction dataset, involving 7,481 gene entities, 8,944 small molecule entities, and 12 interactions. We randomly divided it into a training set (80%), a validation set (10%), and a test set (10%), aiming to predict the relation in an entity pair by the following two steps.

In step 1, we generate a negative set as an equal number of the positive set by closed world assumption, and judge whether an entity pair interacts through an encoder and an FC layer (2 dimensions, representing the probabilities of interacting and non-interacting). In step 2, we predict which relation the interacting entity pair judged by step 1 has through a shared encoder and a FC layer (12 dimensions, each representing the probabilities of 12 interactions). Finally, the overall prediction accuracy is calculated by: $\left[ Acc\left( Interacting \right)*Acc\left( Step 2 \right)+ Acc\left( Non-interacting \right) \right]/2$. The structure of the shared encoder depends on the following five configurations:

1. *NONE*: $\left[ h;t \right]*\Omega_{1}$. Where $\Omega_{1}$ is the concatenate of $1\times2$ vector filters, and embeddings are randomly initialized.
2. *P*: the same structure as *NONE*, but embeddings are initialized by pre-trained embedding result of *S* configuration.
3. *P+C*: $(\left[ \lambda_{C}*t_{c};h;t;\lambda_{C}*h_{c} \right]*\Omega_{1})*\Omega_{2}$. Where $\Omega_{2}$ is the concatenate of $1\times3$ vector filters, and embeddings are initialized by pre-trained embedding result of *S+C* configuration.
4. *P+D*: the same structure as *P+C*, but replace the category part as description part.
5. *P+C+D*: $((\left[ \lambda_{D}*t_{d};\lambda_{C}*t_{c};h;t;\lambda_{C}*h_{c};\lambda_{D}*h_{d} \right]*\Omega_{1})*\Omega_{2})*\Omega_{3}$. Where $\Omega_{3}$ is the concatenate of $1\times3$ vector filters, and embeddings are initialized by pre-trained embedding result of *S+C+D* configuration.

We use the Adam optimizer to minimize the cross-entropy loss function on the training set, finding the optimal combination of hyper-parameters by grid search strategy on the validation set, evaluating our model on the test set. The search scopes of all hyper-parameters are listed: learning rate of step 1 $l\_r\_1\in\{{10}^{-4}, 3\times{10}^{-4}, 5\times{10}^{-4}\}$, learning rate of step 2 $l\_r\_2\in\{{10}^{-4}, 3\times{10}^{-4}, 5\times{10}^{-4}\}$, number of filter $n\_f\in\{10, 20, 30\}$, training epochs of step 1 $ep\_1\in\{100, 200\}$, training epochs of step 2 $ep\_2\in\{100, 200\}$. In addition, batch size is fixed as 10000 and embedding size is fixed as 200.

**References**

Consortium, G.O. (2019) The gene ontology resource: 20 years and still GOing strong. *Nucleic Acids Res*, 47(D1), D330-D338.

Lan, Z. et al. (2019) Albert: a lite bert for self-supervised learning of language representations. *arXiv preprint arXiv:1909.11942*.

Liu, Y. et al. (2019) Roberta: a robustly optimized bert pretraining approach. *arXiv preprint arXiv:1907.11692*.

Mair, P. and Wilcox, R. (2020) Robust statistical methods in R using the WRS2 package. *Behavior research methods*, 52(2), 464-488.

Nickel, M. et al. (2015) A review of relational machine learning for knowledge graphs. *Proceedings of the IEEE*, 104(1), 11-33.

Peters, M. et al. (2018) Deep Contextualized Word Representations. In: *Proceedings of the 2018 Conference of the North American Chapter of the Association for Computational Linguistics: Human Language Technologies*. pp.

Yang, Z. et al. (2019) XLNet: Generalized autoregressive pretraining for language understanding. *arXiv preprint arXiv:1906.08237*.
